# Entomological indices of malaria transmission in Chikhwawa district, Southern Malawi

**DOI:** 10.1186/1475-2875-11-380

**Published:** 2012-11-21

**Authors:** Themba Mzilahowa, Ian M Hastings, Malcolm E Molyneux, Philip J McCall

**Affiliations:** 1 Liverpool School of Tropical Medicine, Liverpool, Pembroke Place, UK; 2 Malawi-Liverpool Wellcome Trust Clinical Research Programme, Blantyre, Malawi; 3 Malaria Alert Centre (MAC), P/ Bag 360, Chichiri, Blantyre 3, Malawi

**Keywords:** Malaria, Africa, Malawi, *Plasmodium*, *Anopheles*, *falciparum*, *Malariae*, *Gambiae*, Transmission, EIR

## Abstract

**Background:**

Although malaria is highly prevalent throughout Malawi, little is known of its transmission dynamics. This paper describes the seasonal activity of the different vectors, human biting indices, sporozoite rates and the entomological inoculation rate in a low-lying rural area in southern Malawi.

**Methods:**

Vectors were sampled over 52 weeks from January 2002 to January 2003, by pyrethrum knockdown catch in two villages in Chikhwawa district, in the Lower Shire Valley.

**Results:**

In total, 7,717 anophelines were collected of which 55.1% were *Anopheles gambiae sensu lato* and 44.9% were *Anopheles funestus.* Three members of the *An. gambiae* complex were identified by PCR: *Anopheles arabiensis* (75%) was abundant throughout the year, *An. gambiae s.s.* (25%) was most common during the wet season and *Anopheles quadriannulatus* occurred at a very low frequency (n=16). *An. funestus* was found in all samples but was most common during the dry season.

*Anopheles gambiae s.s.* and *An. funestus* were highly anthropophilic with human blood indices of 99.2% and 96.3%, respectively. *Anopheles arabiensis* had fed predominantly on humans (85.0%) and less commonly on cattle (10.9%; 1.2% of blood meals were of mixed origin). *Plasmodium falciparum* (192/3,984) and *Plasmodium malariae* (1/3,984) sporozoites were detected by PCR in *An. arabiensis* (3.2%) and *An. funestus* (4.5%), and in a significantly higher proportion of *An. gambiae s.s.* (10.6%)(p<0.01). All three vectors were present throughout the year and malaria transmission occurred in every month, although with greatest intensity during the rainy season (January to April). The combined human blood index exceeded 92% and the *P. falciparum* sporozoite rate was 4.8%, resulting in estimated inoculation rates of 183 infective bites/ person per annum, or an average rate of ~15 infective bites/person/month.

**Conclusions:**

The results demonstrate the importance of *An. gambiae s.s., An. arabiensis* and *An. funestus* in driving the high levels of malaria transmission in the south of Malawi. Sustained and high coverage or roll out of current approaches to malaria control (primarily insecticide-treated bed nets and indoor residual house spraying) in the area are likely to reduce the observed high malaria transmission rate and consequently the incidence of human infections, unless impeded by increasing resistance of vectors to insecticides.

## Background

Malaria is highly endemic and prevalent throughout Malawi, with two thirds of the total population of 12 million people at direct risk of the infection [1,2]. Over 18% of hospital deaths of children less than five years old, and over one third of all outpatient visits, are attributed to malaria [1], the most highly prevalent parasite species being *Plasmodium falciparum*. While there has been substantial research in Malawi on the pathology and chemotherapy of malaria, little is known of malaria transmission dynamics and of the vectorial roles of various anopheline species known to occur in Malawi.

Malaria transmission in Africa is dominated by the *Anopheles gambiae* species complex and the *Anopheles funestus* group of mosquitoes. Studies on malaria transmission in Malawi began in 1911, when two vectors were identified: *An. funestus* which was the most common, and *An. gambiae s.l.* (referred to as *Anopheles costalis*) [3]. Later studies by Lamborn [4] and Berner [5] recorded that while *An. funestus* was abundant throughout the year, *An. gambiae s.l.* was found only in the wet season. In 1992, Hawley *et al.*[6] surveyed districts in the southern (Nsanje and Mangochi) and central (Dowa) regions of Malawi over a four month period in dry and wet seasons. They confirmed the presence of *An. funestus* and *A. gambiae s.l.* and, for the first time, identified both *An. gambiae sensu stricto* (*s.s.*) and *Anopheles arabiensis* in all three sites. Spiers *et al.*[7] recorded *An. gambiae s.s.*, *An. arabiensis, Anopheles merus* and *Anopheles quadriannulatus* (the latter two species in very low numbers); *An. arabiensis* was the predominant anopheline species, comprising over 80% of adult collections. *Plasmodium falciparum* sporozoites have been detected in *An. gambiae s.s.*, *An. arabiensis* and *An. funestus*[6,8] and all three mosquitoes have been shown also to be vectors of *Wuchereria bancrofti* in Malawi [9]. The absence of more comprehensive transmission data for Malawi remains an obvious knowledge gap [10], particularly in an era when reducing transmission is increasingly recognized as an important component of malaria control and a necessary step toward eventual elimination of the infection [11].

Malawi’s malaria control programme is supported by the Global Fund and the US government’s President’s Malaria Initiative (PMI). An initial aim was to reach universal coverage with indoor residual spraying (IRS) by 2011 [12], since such a strategy has proved to be highly effective in some settings [13]. Any optimism must, however, be tempered by reports of resistance to pyrethroids in populations of both the *An. gambiae* and *An. funestus* complexes in southern Malawi [14,15].

To provide basic data on the entomological indices of malaria transmission in southern Malawi, we undertook a study on malaria transmission in the Lower Shire Valley, an area of intense year round malaria transmission. Insight into the genetic structure of *P. falciparum* using data derived from infections in vectors collected from that study has already been reported [16], and here we describe the relative importance of different mosquito species in transmission, their human biting rates and the entomological inoculation rate for malaria in a rural area in southern Malawi.

## Methods

### Study area

The study was carried out in Chikhwawa district (16° 1' S; 34° 47' E), in the Lower Shire Valley, southern Malawi. Based on hospital in-patient records, this area was assumed to have perennial malaria transmission. The area has been a focus of studies investigating malaria-related anaemia, *P. falciparum* genetics and lymphatic filariasis [9,17-19]. Chikhwawa is approximately 70m above sea level, divided throughout its length by the Shire River (the largest river in Malawi) and prone to flooding in the wet season. The area has a tropical climate with a mean annual temperature of 26°C, a single wet season from November to April, and annual rainfall of approximately 770 mm (Malawi Meteorological Office, Chileka, Blantyre). There are extensive rice and sugarcane irrigation schemes.

Following consultation with the District Environmental Health Officer (DEHO) and on the basis of accessibility by road and similarity to other villages in the area, two study villages were selected: Chipula (15° 59' 33" S; 34° 47' 22" E) to the west of the Shire River and north of Chikhwawa District centre (with 109 households), and Kela (16° 02' 72" S; 34° 50' 52" E) (with 117 households) situated to the east of the Shire river. At both villages, the human population lived in thatched brick homes and engaged in subsistence agriculture. Two crops of maize were grown per year. Fishing was common, particularly in Kela, which is situated on the edge of a lake. Some households owned cattle (approximately 50 and 20 animals at Chipula and Kela, respectively) or goats, the majority of which were held in large communal kraals overnight.

At the time of the study, organized vector control in this area had not yet been implemented; few households used insecticide-treated bed nets and indoor residual spraying or other approaches were not in use.

### Mosquito collection

Sampling of adult anopheline mosquitoes was carried out weekly over a period of 52 weeks, from January 2002 to January 2003. Permission to work in the villages was first sought from the village chiefs and householders during community sensitization meetings. Later and during each visit, collections were only carried out after the household owner had provided their free and informed consent to do so. All households were listed at the beginning of the study. Microsoft Excel was used to randomly select individual households, with replacement. To avoid excessive intrusion into the participants’ homes, three new houses were selected at each subsequent visit and a house could be re-visited only after a month. The right to refuse or withdraw at any time was respected. Where permission to enter a house was refused, an alternative house in the immediate proximity was selected.

Collection of mosquitoes was carried out between 6:00 a.m. and 8:00 a.m. Preparation of houses and mosquito collection followed standard procedures [20]. The owners were requested to refrain from sweeping until after collection, and all food items, cooking utensils and water were moved outside during the exercise. White sheets were laid in the rooms to cover the floors, beds and other flat surfaces. The interior space of the house was then sprayed with a pyrethroid-based household insecticide aerosol ‘Doom’ (Dichlorvos, Tetramethrin and d-Phenothrin; Robertsons Homecare Ltd, South Africa) [20]. The eaves and windows were sprayed simultaneously on the outside. The house was exited and doors closed. The sheets were removed for inspection after 10–15 minutes.

All mosquitoes were collected and placed in petri dishes lined with a damp filter paper. Mosquito samples from the 3 houses were kept in separate petri dishes and labeled accordingly. Petri dishes were put in a cooler box and transported to the laboratory in Blantyre for processing.

### Mosquito analyses

Freshly collected anophelines were identified initially using a morphological key [21,22]. Mosquito blood meals were taken from a sub-sample of up to 20 freshly fed mosquitoes per house for subsequent host identification, by crushing individual abdomens onto Whatman® filter papers (No. 3; 110 mm diameter), that were stored in plastic bags containing silica gel until analysis. Abdomens of remaining mosquitoes were then removed and dissected to examine midguts for oocysts [16]. Heads and thoraces of all collected mosquitoes were stored in 1.5 μl eppendorf tubes and stored over silica gel for subsequent examination for *Plasmodium spp*. sporozoites.

### Identification of species within the *An. gambiae species complex*

*Anopheles* cytospecies were identified using ribosomal DNA extracted with LIVAK lysis buffer from individual mosquitoes followed by DNA amplification by the standard rDNA-PCR method [23,24]. If the initial PCR test failed to amplify for a sample, it was repeated twice until successful amplification occurred, or was scored as unknown. Reactions included negative and positive controls. All reactions were carried out using the GeneAmp® PCR System 2700 (Applied Biosystems, UK).

### Analysis of blood meals

A direct enzyme-linked immunosorbent assay (ELISA) was used to identify the source of mosquito blood meals [25]. Each sample was analysed by two separate assays: one for human blood and one for bovine blood. Positive results were read visually, 30 minutes after adding the substrate ABTS (2,2′ azino-bis 3-ethylbenzthiazoline-6-sulphonic acid) and hydrogen peroxide (Kirkegaard and Perry Laboratories, USA) in a 1:1 mixture ratio. A sub-sample of 100 positive specimens was re-run for each test to confirm the test.

### Detection of *Plasmodium* spp. sporozoites

*Plasmodium* spp. sporozoites were detected and identified by PCR on DNA extracts from heads and thoraces of individual female mosquitoes [26]. DNA was extracted [23] and diluted to 10% in water for optimal PCR amplification. PCR reactions were performed using the Applied Biosystems, GeneAmp® PCR System 2700 thermocycler. The cycling parameters were: step 1, 95°C for 5 minutes; step 2, 94°C for 1 minute, annealing at 58°C for 2 minutes, extension at 72°C for 2 minutes; step 3, final extension at 72°C for 5 minutes.

### Climate data

**Figure 1:**
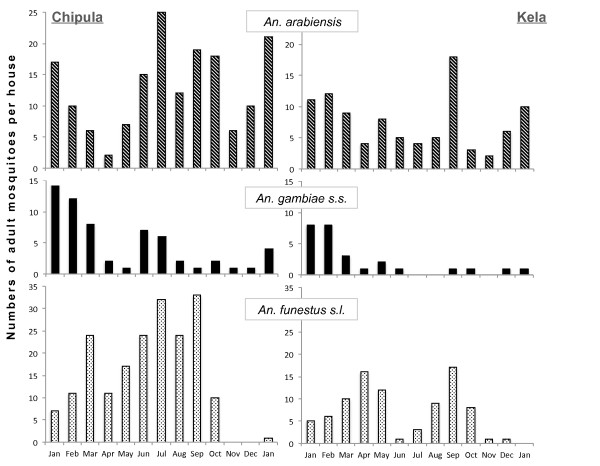
**Monthly rainfall (black bars), maximum temperature (dotted line) and minimum temperature (dashed line) measured at Kasinthula weather station in Chikhwawa, close to the study sites and covering the same period as the data presented in Figure**1**.**

Climate data measured at Kasinthula irrigation project (16° 5' S, 34° 49' E; the nearest weather station to the study sites) was obtained from the central meteorological offices at Chileka International Airport in Blantyre. Monthly mean rainfall, and daily mean, maximum and minimum temperatures were recorded (Figure 1).

### Data analyses

Data were analysed using SPSS version 12.0.1. Proportions were compared using Chi-Square test, or Fisher’s Exact test where appropriate. The entomologic inoculation rate (EIR) was calculated from the mean numbers of blood-fed mosquitoes (number of mosquitoes divided by the number of house occupants, multiplied by the human blood index for each mosquito species) multiplied by the sporozoite rates. The estimated annual EIR was the sum of the monthly means for the three vectors.

### Ethical issues

Ethical permission for the study was provided by the Research Ethics Committees of the College of Medicine Blantyre (COMREC) and the Liverpool School of Tropical Medicine. Permission to work in specific villages was granted by each village chief following an initial briefing meeting at which the nature and objectives of the study were explained to all members of the community in the local language, Chichewa. Written informed consent was obtained at the beginning of the study. On the day of mosquito sampling, the purpose of the work was again explained to each householder, and permission to enter the house was sought.

## Results

### Anopheline species composition

A total of 7,717 anopheline mosquito adults were collected from houses in the two study villages between January 2002 and January 2003. Only members of the *An. gambiae* species complex (n=4,253; 55.1%) and *An. funestus* group of mosquitoes (n=3464, 44.9%) were found (Table 1). The proportions of *An. gambiae s.l.* were slightly higher than *An. funestus s.l* at both study villages (χ^2^ = 6, df = 1, p<0.013). *An. gambiae s.l.* comprised 57.2% (n=1,407) and *An. funestus* 42.8% (n=1,054) of the anophelines collected at Chipula, the equivalent proportions for Kela being 54.1% (n=2,846) and 45.9% (n=2,410).

**Table 1:** **Total numbers of*Anopheles*adult female mosquitoes collected by pyrethrum knockdown catch from houses in two villages, Chipula and Kela, in the lower Shire valley, Malawi, between January 2002 and January 2003**

**Species**	**Chipula**	**Kela**	**Total**
*An. funestus*	1054	2410	3464
*An. gambiae s.l.*	1407	2846	4253
*An. arabiensis*	831 (76.5%)	1642 (74.8%)	2437
*An. gambiae s.s.*	248 (22.8%)	547 (24.9%)	795
*An. quadriannulatus*	8 (0.7%)	6 (0.3%)	14

A total of 3,410 individual mosquitoes of the *An. gambiae* complex were identified to species level by PCR and three species were identified: *An. arabiensis* (n=2,473), *An. gambiae s.s.* (n=795) and *An. quadriannulatus* (n=14). The proportions of these species were similar at both villages: *An. arabiensis* comprised 76.5% (n=831), *An. gambiae s.s.* 22.8% (n=248) and *An. quadriannulatus* 0.7% (n=8) at Chipula, and 74.8% (n=1,642), 24.9% (n=547) and 0.3% (n=6) respectively at Kela.

No formal identifications of the *An. funestus* were carried out in this study. However, all 30 specimens in a sample of this species (collected from Kela village) were identified as *An. funestus s.s.* (Besansky, *pers. communication*) [27].

### Seasonal variation in species abundance

**Figure 2:**
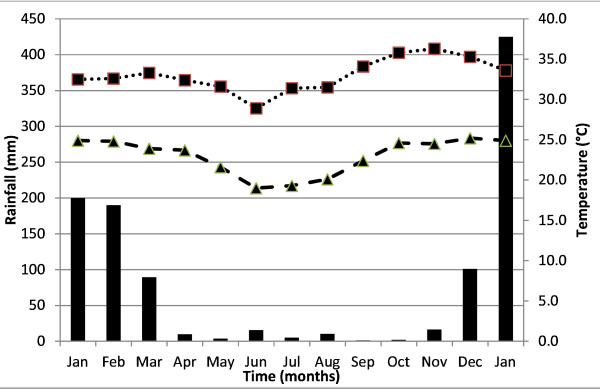
**Monthly abundance of*An. arabiensis, An. gambiae s.s.*and*An. funestus*from Chipula and Kela in the lower Shire valley, as measured by pyrethroid knockdown catch (Jan 2002 - Jan 2003).**

The three most common anophelines, *An. funestus, An. arabiensis* and *An. gambiae s.s.,* were present throughout the entire collection period, although the relative abundance varied markedly in different months (Figure 2).

*Anopheles arabiensis* was the predominant species in all catches, in Chipula ranging from 59% (n=121/205) of the total monthly catch (*i.e.* total number of all anopheline mosquitoes caught) in January 2002 to 97% (n=34) in August 2002, and in Kela ranging from 44% (n=52) in March 2002 to 93.6% (n=204) in September 2002 for Chipula and Kela respectively.

*Anopheles gambiae s.s.* proportions in Chipula varied from 2.9% (n=1) in August 2002 to 42% (n=88) in January 2002, and in Kela varied from 6.4% (n=14) in September 2002 to 56% (n=66) in March 2002. Unlike *An. arabiensis* and *An. funestus*, more adults of *An. gambiae s.s.* were found during the wet season when both minimum and maximum temperatures were also high (Figure 1).

The proportions of adult female *An. funestus* in monthly catches ranged from 76% (n = 193) in April 2002 to 2.9% (n = 4) in January 2003 in Chipula, and from 74% (n = 129) in April 2002 to 2% (n = 2) in November & December 2002 in Kela (Figure 2). At both sites *An. funestus s.l.* numbers were greatest during the drier months (March to September) when temperatures were also slightly lower (Figure 1).

### Sources of mosquito blood meals

A total of 2,300 mosquito blood meal specimens were collected for identification, a random sample of which (n=883; 38.4%) were analysed by ELISA to identify the source of the ingested blood (Table 2). Humans were the most common blood meal source for all mosquitoes (92.8%; n=819), with only 5.3% (n=47) feeding on cattle. Only 5/883 (0.6%) blood meals were from both hosts. Twelve samples (1.4%) were negative in both ELISAs, either because the reactions failed or possibly because the blood was derived from other hosts. A random sample of 100 specimens that tested positive for human blood was re-tested with similar results.

**Table 2:** **Identity of blood meals from*An. arabiensis, An. gambiae s.s.*and*An. funestus*adult female mosquitoes collected by pyrethrum knockdown catch in the lower Shire valley (Jan 2002 - Jan 2003)**

**Species**	**Blood meal source**				
	**No. tested**	**Bovine**	**Human**	**Bovine/Human**	**Other**
*An. arabiensis*		37 (10.9)	289 (85.0)	4 (1.2)	10 (2.9)
*An. gambiae s.s.*	246	1 (0.4)	244 (99.2)	1 (0.4)	0
*An. funestus*	297	9 (3.0)	286 (96.3)	0	2 (0.7)
**Total**	**883**	**47 (5.3)**	**819 (92.8)**	**5 (0.6)**	**12 (1.4)**

Blood meals were screened twice by ELISA for either human blood or for bovine blood, and blood meals that were negative in both screens were classed as ‘other’.

**Figure 3:**
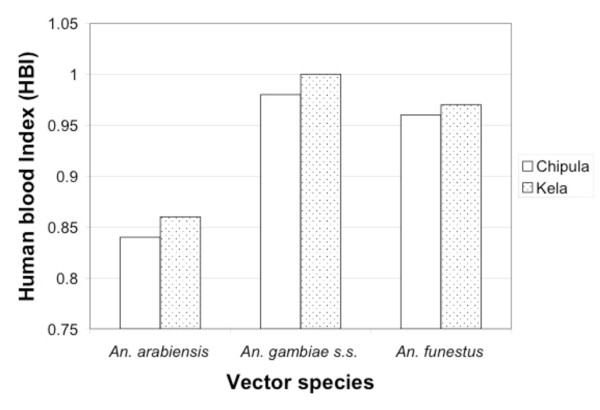
**Human blood indices (HBI) for the three malaria vectors in two villages, Chipula and Kela, in the lower Shire valley, Malawi (Jan 2002 - Jan 2003).** Numbers of blood meals analysed were: *An. gambiae s.s.*, 115, 131; *An. arabiensis* 160, 180; *An. funestus* 122, 175; from Chipula and Kela respectively.

Figure 3 shows the human blood indices of the three vector species at both sites. Indices were very high for *An. gambiae s.s* (0.983 and 1) and for *An. funestus* (0.959 and 0.969) and slightly less for *An. arabiensis* (0.838, 0.861). In all cases, significantly more blood meals were taken from humans than from cattle, although this preference was significantly lower in *An. arabiensis* than in the other two species (Fisher’s exact test, df = 6, p<0.000).

### *Plasmodium sp.* oocyst rates

A total of 7,717 adult female anophelines were dissected and oocysts were found on the midguts of 100 (1.3%). Significantly more mosquitoes (84%) infected with oocysts were collected from Kela village (90.5%; n = 656) than from Chipula (9.5%; n = 69) (χ^2^ = 11.78; *P*<0.001). The estimated number of oocysts per midgut varied from 1 to 63 and the mode was 1 oocyst per midgut; data were heavily skewed with a mean of 6 and a very large variance of 66, and were described best by a negative binomial distribution. Oocyst rates were significantly higher in *An. funestus* (1.79%; n=3519) than in *An. gambiae s.l* (0.88%; n=4198). The mean oocyst load was slightly higher for adult female *An. gambiae s.l* (7.9; 95% CI 4.3 – 11.5) was slightly but not significantly higher than *An. funestus* (6.9; 95% CI 4.6 – 9.2). The dissected oocysts were processed and used for a related study, as reported previously [16].

### *Plasmodium spp.* sporozoite rates

Screening of 3,958 mosquitoes (2,315 *An. arabiensis,* 718 *An. gambiae s.s.* and 925 *An. funestus*) for *Plasmodium spp.* sporozoites using PCR (Table 3), identified 192 (4.85%) as infected. All carried *P. falciparum* alone, except for a single *An. gambiae s.s.* that was infected with both *P. falciparum* and *Plasmodium malariae.* No *Plasmodium ovale* parasites were detected*.* Infective mosquitoes were found in every month with highest prevalence occurring in April. *Anopheles gambiae s.s.* had the highest year-round sporozoite rate of 10.6%, significantly greater than both *An. funestus* (4.5%) and *An. arabiensis* (3.2%) (p<0.05). The inoculation rates of *An. arabiensis, An. gambiae s.s* and *An. funestus* were 5.82, 6.71 and 2.33 infective bites/person/month at Chipula and 5.45, 8.09 and 2.14 infective bites/person/month at Kela. The community in this area therefore received an estimated average of 183 infective bites/ person/ annum. In both villages, *An. gambiae s.s* was the most important malaria vector, being responsible for 44% and 52% of the transmission at Chipula and Kela respectively.

**Table 3:** **The total numbers of mosquitoes examined and sporozoite rates for*An. arabiensis*,*An. gambiae s.s*. and*An. funestus*collected by pyrethrum spray catch from Chipula and Kela villages in the lower Shire valley Malawi, between January 2002 and January 2003**

	**Total**	**Chipula village**			**Kela village**		
		**No. tested**	**No. positive**	**Sporozoite rate (95% CI)**	**No. tested**	**No. positive**	**Sporozoite rate (95% CI)**
*An. arabiensis*	2315	792	35	4.4 (2.9 – 5.8)	1523	39	2.6 (1.8 – 3.4)
*An. gambiae s.s.*	718	219	27	12.3 (10.2–4.4)	499	49	9.8 (8.2–11.5)
*An. funestus*	925	411	21	5.1 (0.8 – 9.5)	514	21	4.1 (1.5 – 6.7)
**Total**	**3958**	**1422**	**83**	**5.8 (4.6-7.0)**	**2536**	**109**	**4.3 (3.5 – 5.1)**

## Discussion

The results demonstrate the importance of all three of the abundant anophelines in the region, *An. gambiae s.s, An. arabiensis* and *An. funestus,* as malaria vectors driving the high levels of malaria transmission in the south of Malawi. All three vectors were present throughout the year and malaria transmission occurred in every month. The combined human blood index exceeded 92% and the *P. falciparum* sporozoite rate was 4.8%, resulting in inoculation rates of 183 infective bites/ person per annum, or a monthly rate of ~15 infective bites/ person.

This EIR is higher than some previous estimates from Malawi where annual values of only 46.1 and 27.7 infective bites/ person/ year were recorded for Mangochi and Nsanje respectively [6] but close to those recorded in Nkhotakota in the central region of Malawi where Chiphwanya (2003) recorded sporozoite rates of 6.5%, 5.2% and 4.5% in *An. gambiae, An. funestus* and *An. arabiensis* respectively, an average sporozoite rate of 5.4% in all three vectors [8].

These rates are higher than in neighbouring Mozambique, where inoculation rates of 20 and 27 infective bites/ person/ year were recorded [28,29]. Indeed, the EIRs in this study are closer to those recorded further north in Tanzania or Kenya where values of this order or higher are common [30]. These high rates may be due, in part at least, to the use of the highly sensitive PCR method to screen mosquitoes for sporozoites as compared with microscopic examination [31]. Previously in Malawi, Chiphwanya [8] recorded comparable rates to these using ELISA. Such problems when comparing EIRs from different localities or dates have been noted previously [30,32]. Notably, the oocyst rate (1.3%) is lower than the sporozoite rate (4.85%). While PCR may have been highly sensitive in detection of sporozoites, it is also likely that detection of oocysts by microscopy failed to detect light infections at this stage of development, though this alone would be unlikely to account for the discrepancy between the rates.

Thus, the high EIR reported here may derive from a combination of factors, both genuine (the presence of three efficient vector species and the absence of vector control, the low availability of hosts other than humans) and potentially confounding (sensitivity of PCR in detection of Plasmodium sp. DNA, collection of endophilic anthropophilic mosquitoes only). Conversely, relying on the pyrethrum knockdown catch method meant that the study sampled only indoor resting mosquitoes. Exophilic mosquitoes would not have been caught, an important consideration if those mosquitoes were also partly zoophilic, and one that would have contributed to an underestimate of EIR. Thus in parts of Kenya, HBI values for *An. arabiensis* can be as low as 0.22 [33] or even 0.095 [34]. It is somewhat reassuring that HBI values comparable to ours (0.85) have been recorded in similar localities in neighbouring Zambia (0.872, 0.923, 0.94/0.96) [35-37] and Mozambique (0.985) [29]. One Zambian study found that although the relative proportions of the *An. arabiensis* population exhibiting endo or exophilic behaviour varied between different years, the HBI values remained high [37]. These various studies are consistent with the data presented here and indicate a strong anthropophilic tendency in *An. arabiensis* populations in southern Malawi and neighbouring areas.

The three species: *An. funestus*, *An. arabiensis* and *An. gambiae s.s.* fed predominantly on humans (92.8%) as confirmed by ELISA test results. The other blood meals were taken from cattle, and were mainly in *An. arabiensis* (10.9%). Few feeds (1.2%) were of mixed origin of these two hosts. Although cows were available in both villages, they were not common (only 70 in total in both villages) and were herded into open corrals (or *bomas*) at night; we did not collect mosquitoes from there, although it might be expected that numbers of *An. arabiensis* feeding on those animals could be very high. However, any potential zooprophylactic effect was not sufficient to prevent *An. arabiensis* from feeding on humans at a frequency that resulted in a sporozoite rate of 3.2% in the endophilic proportion of its population.

The high inoculation rates reported here result, at least in part, from the presence of three competent vector species that are present all year round permitting transmission in every calendar month. The importance of *An. gambiae s.s.* and *An. funestus* in Malawi is recognised but the demonstration of high levels of transmission by *An. arabiensis* is in sharp contrast with current reports that it does not play a significant role in malaria transmission [12]. In fact, *An. arabiensis*, along with *An. funestus,* are important in extending the duration of the malaria transmission period well beyond the wetter months. The most efficient vector, *An. gambiae s.s* was highly infective during the wet season *i.e.* between January and May 2002 with peak sporozoite rates experienced in April 2002 (late rainy season; results not shown) when high numbers of this species were found.

High EIRs, like those reported here, are associated frequently with correspondingly high prevalence rates of parasitaemia in human populations [38]. High inoculation rates can have important implications for malaria epidemiology, including the likely age at first malaria infection and the clinical disease pattern. Under high transmission intensity, children are likely to be infected at an early age, as demonstrated in a study in Mangochi in central Malawi [39] where malaria transmission is also intense, in which 60-80% of infants under 10 months of age in a cross-sectional survey were parasitaemic, the prevalence varying with season. When a high EIR causes *P. falciparum* infections to occur early in life, severe anaemia is common and is the predominating form of severe malaria among infants and toddlers [40], while those surviving to adulthood have acquired partial immunity and rarely suffer severe complications from *P. falciparum* infections.

The biggest challenge in malaria control is to reduce the observed high EIRs in order to achieve reductions in human infection rates sufficient to reduce the burden of disease. Simple but effective technologies directed against vectors – long-lasting insecticide-impregnated bed nets (LLINs) and indoor residual spraying (IRS) – can dramatically reduce malaria transmission intensity. The high levels of indoor biting reported here indicate that, subject to the vectors remaining susceptible to pyrethroid and other insecticides [15,41-44], the currently planned [12] roll-out of both LLINs and IRS is likely to reduce the EIR in the Lower Shire valley. The recently reported success achieved further north in the Nkhotakhota district of Malawi supports this prospect [13]. As elsewhere in Africa, the relative importance of exophilic *An. arabiensis* as a malaria vector is likely to increase gradually as a consequence of control measures that reduce the contribution of endophilic mosquitoes, and the development of new measures to combat these more elusive vectors will become increasingly important.

## Competing interests

The authors declare that they have no competing interests.

## Authors’ contributions

Conceived, designed and managed the study protocol: PJM, TM, IH, MM. Undertook the field collections and laboratory analyses: TM. Interpreted, analysed data and wrote the paper: TM, PJM, IH. All authors read, edited and approved the final version of the manuscript.
